# Discrimination of topsoil environments in a karst landscape: an outcome of a geochemical mapping campaign

**DOI:** 10.1186/s12932-019-0065-z

**Published:** 2020-01-04

**Authors:** Ozren Hasan, Slobodan Miko, Nikolina Ilijanić, Dea Brunović, Željko Dedić, Martina Šparica Miko, Zoran Peh

**Affiliations:** Croatian Geological Survey, Sachsova 2, P.O. Box 268, 10000 Zagreb, Croatia

**Keywords:** Geochemical mapping, Soils, Compositional data, Discriminant function analysis, Karst, Croatia

## Abstract

The study presented in this work emerged as a result of a multiyear regional geochemical survey based on low-density topsoil sampling and the ensuing geochemical atlas of Croatia. This study focuses on the Dinaric part of Croatia to expound the underlying mechanisms controlling the mobilities and variations in distribution of potentially harmful elements as observed from different environmental angles. Although serious environmental degradation of the vulnerable karst soil landscapes was expected to occur chiefly through the accumulation of various heavy metals, the most acute threat materialized through the soil acidification (Al-toxicity) affecting the entire Dinaric karst area. This picture surfaced from the analysis of all three investigated discriminant function models employing the abovementioned environmental criteria selected autonomously with respect to the evaluated soil geochemistry, namely, geologic setting, regional placement and land use. These models are presented by not only the characteristic discriminant-function diagrams but also a set of appropriate mathematically derived geochemical maps disclosing the allocations of potential threats to the karst soil landscapes posed by soil acidity.

## Introduction

As soon as the Geochemical Atlas of Croatia (GAC) was published at the end of the last decade [27], it became obvious that the search for the regional geochemical background (at least on the territory of a single country) is its primary goal. The formation of systematic and relational geochemical GIS databases as the secondary goal would open a number of new exploratory avenues to be covered in the following years. A strong signal suggesting that the in-depth analysis of various variables (state factors) involved in the process of soil formation and development is essential for understanding the soil geochemistry had echoed from the first multi-element geochemical map of Croatia [28]. This thematic map, based on the posterior classification probabilities, employed the regional division (faithfully epitomizing contrasting bedrock lithology) as an independent grouping criterion in discriminant function analysis (DFA) of Croatian topsoil (interval 0–25 cm) geochemical data. In recent times, the probability maps, albeit originally designed for the purpose of petroleum prospecting [29], proved very useful in urban geochemical studies dealing with soil complexity on a local scale [65]. However, on a regional scale (GAC), this type of map clearly distinguished the Dinaric (DIN) from the Pannonian (PAN) part of the Croatian territory on account of the extremely high mean classification rate of 94% for the total set of data (low-density regional survey with 2521 samples in a regular 5 × 5 km grid) whereby samples were a priori classified as pertaining to either the DIN or PAN group [28]. Accordingly, the two regions, each presented as a single geodynamic unit with its own set of soil-forming factors and local disturbances, have been considered distinct entities, suggesting separate studies as the most effective approach to further geochemical and environmental investigations. Out of this dichotomy, the Dinaric region (as any karst area) was brought into sharp focus owing to its characteristic carbonate lithology, which provides the geochemical basis for an extremely fragile karst ecosystem whose soil cover is frequently exposed to erosion and pollution because of improper land use [24]. Unable to cope with specific hazards and impacts caused by neglectful human activity, the vulnerable soil landscapes on the Adriatic coast and its neighbouring mountainous area show the symptoms of increasing environmental degradation. Most of the changes that affected soils arose from growing tourism and expansion of urban areas, recent/collapsed industrial activity (including mining and quarrying), and deforestation caused either by natural (e.g., freezing rains) or anthropogenic (e.g., acid rains) effect (see [68]). Necessarily, it became imperative for future research activities, especially in this area, to fathom the various environmental factors involved in the processes described above because notwithstanding the growing intensity and scale of their use and abuse, the soils stay firmly ingrained at the foundations of human life [39] necessitating sustainable environmental management.

All things considered, the main objective of this study is to investigate the factors responsible for the characteristic geochemical signature of the modern soils developed over the Dinaric karst in the south-western exposures of Croatia. To this purpose, the topsoils collected during the multi-year geochemical mapping campaign (GAC) are examined in light of the various geological and environmental criteria [40–42, 51, 58, 59]. These criteria, as in other geochemical studies on similar problems in the area [34, 52, 53], are exploited in this study as the most revealing avenues through which the processes mentioned above can be most effectively understood. The criteria are autonomous with regard to the soil geochemistry such as the geological (lithological) setting, description of land use, soil types, or geographical position (with climate implications), which provide the most efficient means of a priori arrangement of the soil samples into a number of coherent and exhaustive statistical groups. In the final analysis, DFA is employed as a method of data reduction and organization, generating the models based on geochemical partitioning between the established groups. As mathematical models by their nature, they generate the structural patterns that help describe the behaviour of the observed geochemical data in process-form terms [64], notably in the form of maps—the spatial structural patterns [43].

## Materials and methods

### Description of the study area

**Fig. 1:**
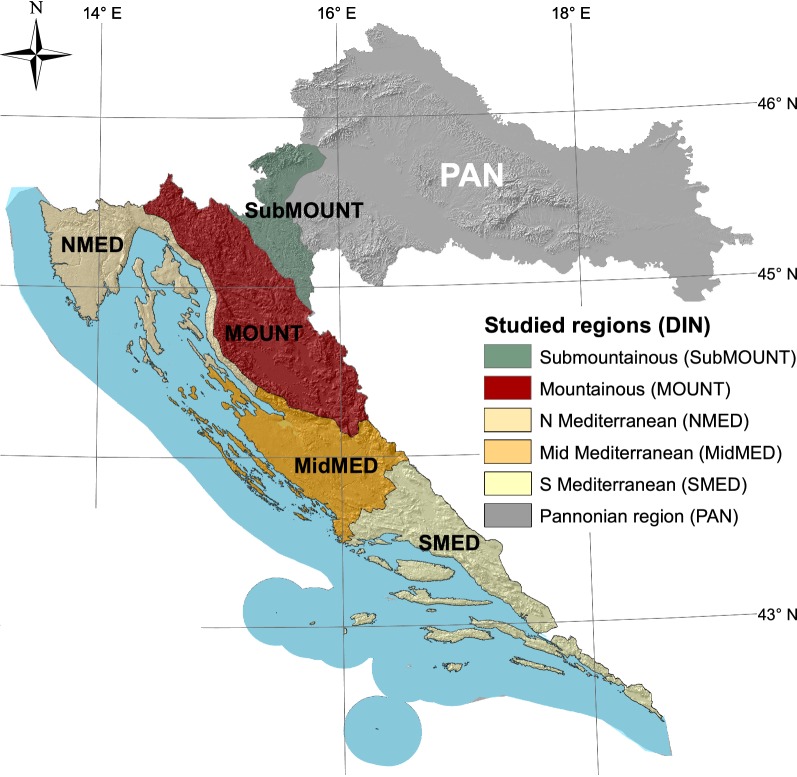
Geographical position of the study area with sampling points (5 × 5 km grid)

Croatia is a Mediterranean and Central European country geographically located between 13.5° and 19.5° eastern longitudes and 42.5° and 46.5° northern latitudes, extending from the vast Pannonian plain across the narrow Dinaric mountain range to the Adriatic coast. Almost half of its territory (46%) is located in its maritime (Adriatic) and mountainous (Dinaric) regions (Fig. 1). As a result, the climate is strongly controlled by relief, ranging from continental temperate in the mountains (Cfc and Dfc types) to Mediterranean and sub-Mediterranean (Csa and Cfa types) along the coastline and in the adjacent hinterland [74]. Although the Dinarides are the most important mountain range (Dinara Mt., 1831 m), only 0.11% of the mountain topography is situated above 1500 m ASL. Nevertheless, the mountain barrier strongly affects the mean annual temperatures and precipitation. Temperatures increase from west to east while precipitation varies inversely with temperatures: mountain areas are characterized by high amounts of precipitation between 1100 and 1940 mm, while average rainfall in the coastal area varies between 855 and 1253 mm [8]. Acid rains are at the heart of the problem in the mountainous zone, particularly in the Velebit Mt. area, affecting the growth of the Dinaric beech-fir forest communities [6, 37].

### Geological setting and soils

**Fig. 2:**
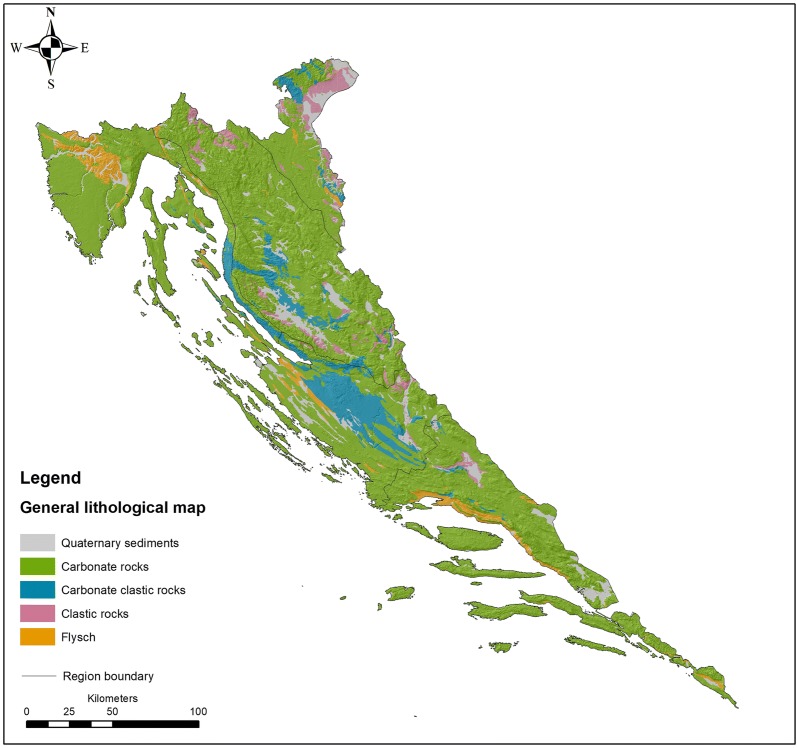
Simplified geological map of the study area

The area of the Croatian karst Dinarides is represented by a thick succession of carbonate rocks deposited between the Late Palaeozoic (Middle Permian) and the Eocene on platforms of different ages, types and palaeogeographic settings (Fig. 2). The evolution of DIN began on an epeiric carbonate platform situated at the northern Gondwana margin with significant deposition of mixed carbonate-siliciclastic sediments during the Permian and mostly siliciclastic deposits in the Early Triassic [72]. The Middle Triassic was marked by the separation of the Adria Microplate and sedimentation of carbonate facies with locally significant volcanoclastic influences. Late Triassic dolomites and limestones represent typical deposits of the large isolated Southern Tethyan mega-platform [72] that experienced rift-induced fragmentation resulting in a number of long-lasting carbonate platforms during the Triassic-Jurassic transition. The largest among these platforms was Adriatic-Dinaric Carbonate Platform (ADCP) consisting of four tectonostratigraphic units: the Dinaric NE unit (Inner Karst), Dinaric SW unit (High Karst), Adriatic NE unit (Dalmatian Karst) and Adriatic SW unit (Istrian Karst) ([33] and references therein). The disintegration of the ADCP, characterized by ramp-type carbonate deposition along the margins and the development of flysch basins, started in the Late Cretaceous, while the Cretaceous-Palaeogene transition was marked by a period of regional emergence involving the entire platform. As dynamic tectonics continued into the Palaeogene, the platform depositional sequences were mostly under control of intense synsedimentary tectonics, sometimes deposited in the ramp-like settings. The final uplift of the entire Dinaric area as a result of collision between the Adriatic and Dinaric segments reached its culmination in the Oligocene–Miocene.

Diverse and complex bedrock geology, climate and relief result in a wide range of soils developed in the DIN landscape. Encompassing the coastal and mountainous areas in the SW part of Croatia with their typical carbonate bedrock, the landscape is dominated by various types of automorphic soils, in particular polygenetic Cambisols (eutric, distric, chromic) developed on dolomite and limestone as well as Leptosols, Regosols, Melanosols and anthropogenic soils on flysch bedrock [7]. In the southern part of the Croatian coastal regions are areas under hydromorphic soils—Fluvisols and Gleysols—especially in the Neretva River valley and in some karst poljes [8, 11].

### Field and analytical procedures

#### Sampling

The locations of the sampling sites and the sampling density were defined by the systematic sampling design according to the ISO 10381-1 and ISO 10381-2 [30, 31] protocols whereby each cell represents the area of 25 km^2^ in a regular grid of 5 × 5 km^2^. This scheme includes 1247 soil sampling sites covering the entire DIN part of the country (Fig. 1). Samples were collected from the centre of each cell within the tolerance circle of 15% around the central cell point. The randomness of sampling sites was defined during a pilot geochemical mapping project related to karst terrains in Croatia, which situated the initial sampling point in the heart of the Istrian Peninsula [57]. Soil samples were taken in the center of the 5 × 5 km cell. Soils were sampled with a plastic spade from five shallow pits on each site in the depth interval 0 to 25 cm. One composite sample was prepared for every sampling location. Detailed soil sampling protocols, statistical methodology, and the choice of sampling cell size are presented in the papers by Pirc et al. [57], Prohić et al. [58, 59], and Miko et al. [40, 42] and finalized by the completion of the GAC [27], together with the laboratory protocols and a detailed field and data handling manual that basically followed the geochemical mapping protocols presented in the report by Darnley et al. [16].

Inasmuch as numerous environmental geochemical studies have shown that the optimal grain size fraction for characterization of the trace element contents of soils and sediments should not exceed 0.180 mm [16, 35, 66], the chemical analysis was carried out on fractions < 0.063 mm.

#### Sample preparation and analysis

The soil samples were dried and homogenized and then dissolved in a mixture of concentrated acids HF-HCl-HNO_3_-HClO_4_. Solutions were analysed by mass spectrometry using a Perkin Elmer Elan 6000 or 9000 ICP-MS [2] for a set of 41 elements: Ag, Al, As, Au, Ba, Be, Bi, Ca, Cd, Ce, Co, Cr, Cu, Fe, Hf, K, La, Li, Mg, Mn, Mo, Na, Nb, Ni, P, Pb, Rb, S, Sb, Sc, Sn, Sr, Ta, Th, Ti, U, V, W, Y, Zn, and Zr. In the process, the recovery of refractory minerals such as cassiterite, wolframite, chromite, spinel, beryl, zircon, tourmaline, magnetite and barite was incomplete after the 4-acid digestion. Moreover, due to the evaporation of HClO_4_, losses of As and Cr were also possible, while silica completely evaporated with HF. Mercury analysis was performed using aqua regia extraction by flameless atomic adsorption spectrometry (FAAS).

The accuracy was controlled by certified geological reference materials, i.e., GXR-2, GXR 5, and SJS-1 soils from the USGS (ACME Labs). The accuracy for most elements analysed in reference soil materials was found in the range of ± 10% of the certified values. The precision was determined by repeated analyses of both certified reference samples and randomly selected soil samples (every 20th sample in the batch) with a resulting average coefficient of variation of approximately 5%.

Analyses of total organic (TOC) and inorganic (TIC) carbon abundances were performed on sieved soil samples with an elemental Thermo Fisher Scientific Soil Flash 2000 NC analyser. To determine carbon in organic form, carbon measurements were carried out after removal of carbonates from the soil. Carbonates were removed by adding an aqueous acid solution (1 M HCl) to soil samples. A subset of samples was checked by XRD diffraction to check whether the dissolution of carbonates was complete. The total inorganic carbon was calculated by the difference between total carbon (untreated sample) and total organic carbon (sample treated with 1 M HCl).

### Statistical processing and map generation

#### The data

The spatial continuous geochemical database of the Dinaric region includes 1459 samples of which the greater part (1247 samples) was collected in a regular grid of 5 × 5 km while the rest was taken from denser grids of 2.5 × 2.5 km and 1 × 1 km, which had been designed for the purpose of closer inspection into the geochemical landscapes of several national parks of Croatia (Brijuni, Plitvice Lakes, Risnjak and Mljet) as well as special karst features such as karst poljes [59]. The latter database was created for special purposes and was not included in the present study. The primary 5 × 5 cell database planned for statistical analysis was formed in the ESRI^®^ ArcInfo™ 10.2.1 GIS software and designed in such a way that each particular sample point was connected to the set of its description data consisting of coordinates, relief, slope, lithology, soil structure, soil texture, environment, potential pollution, organic matter, remarks, colour description (according to Munsell [44]), results of chemical analysis, three levels of Corine Land Cover 2012 categories, geographic region, and soil type. A reduced set of 26 (8 major and 18 trace elements) out of the total set of 41 analysed elements and soil organic carbon (TOC) was selected for further analysis and representation in this work following the recommendations of Darnley et al. [16] that elements having concentrations lower than detection limits in more than 20% of the samples should not be exploited for statistical and mapping purposes. Where measured concentration values were below the detection limit, half the detection limit was used for statistical analysis according to the Guidelines for the FOREGS-EuroGeoSurveys’ Geochemical Baseline Mapping [16, 19, 62].

#### Compositional data analysis

The original dataset contains the suite of 28-part geochemical compositions used routinely in similar previous investigations based on the low-density soil sampling during the geochemical mapping of Croatia (e.g., [28, 42, 51]), namely, Al, As, Ba, Ca, Cd, Co, Cr, Cu, Fe, K, La, Mg, Mn, Na, Nb, Ni, P, Pb, Sc, Sr, Th, Ti, V, Y, Zn, and Zr but also including N and TOC in this case. These variables were selected as input data (predictors) for discriminant function analysis (DFA) in pursuance of the behaviour of potentially toxic elements in the topsoils (soil depth from 0 to 25 cm) developed on the Croatian karst. Descriptive statistics for the whole dataset—min, max, median, Q1 and Q3 quartiles, median absolute deviation (MAD) and geometric mean (g)—are summarized in Table 1 showing, however, information that is suitable only for comparison purposes since the data displayed represent relative rather than absolute values. A notorious truth that soil geochemical data represent a typical example of compositional data (CoDa) should effectively preclude their use in the raw form in any statistical analysis [22]. The nature of CoDa involves the mathematical peculiarity that all variables (component parts) in each individual case (analysed sample) are always positive and constrained to a constant sum defined a priori as 100%, 10^6^ ppm, or 1.0. By virtue of the unit-sum constraint, CoDa can be naturally displayed only in the restricted sample space (compositional space) known as simplex and consisting of D parts or components (geochemical variables). A set of D-part composition (S^D^) occupies a restricted part (from zero to, say, 100%) of a D-dimensional real space (R^D^), forming a subset of its vectors [12, 13, 46]. The principles of the simplex as the natural sample space for compositional data are conveyed through the following expression [12, 47]:

**Table 1:** Descriptive statistics for raw (compositional) geochemical data

Element	Min	Q1	Med	Q3	Max	MAD	g
Al (%)	0.9	6.48	7.45	8.48	14.04	1.010	7.087
As (ppm)	2.2	12.0	16.0	22.0	105.0	5.000	15.133
Ba (ppm)	35.0	253.0	306.0	360.0	840.0	53.000	289.491
Ca (%)	0.08	0.58	0.97	2.07	28.73	0.051	1.224
Cd (ppm)	0.1	0.4	0.9	1.5	15.5	0.6000	0.750
Co (ppm)	2.0	13.0	17.0	20.0	43.0	3.000	15.682
Cr (ppm)	15.0	84.5	107.0	132.0	443.9	23.100	105.720
Cu (ppm)	6.0	23.0	31.1	40.8	429.0	8.700	31.406
Fe (%)	0.43	3.24	3.9	4.57	8.02	0.660	3.714
K (%)	0.18	1.07	1.29	1.53	3.79	0.230	1.247
La (ppm)	4.0	40.0	49.0	57.0	185.0	8.000	46.006
Mg (%)	0.16	0.54	0.7	0.87	10.47	0.16	0.755
Mn (ppm)	96.0	711.0	957.0	1223.0	3839.0	254.0	895.123
Na (%)	0.048	0.276	0.419	0.6	1.78	0.162	0.393
Nb (ppm)	1.0	10.1	13.0	17.0	30.0	3.000	12.794
Ni (ppm)	7.0	50.0	68.0	88.0	289.0	18.200	64.618
P (%)	0.015	0.047	0.061	0.085	0.684	0.017	0.065
Pb (ppm)	10.0	33.0	43.0	54.0	177.0	10.200	41.715
Sc (ppm)	1.0	10.0	12.0	13.0	115.0	2.000	11.045
Sr (ppm)	22.0	72.0	84.0	98.0	588.0	13.000	87.551
Th (ppm)	2.0	12.0	14.4	17.2	29.7	2.600	13.672
Ti (%)	0.05	0.374	0.428	0.473	0.94	0.050	0.403
V (ppm)	9.0	111.0	137.0	167.0	473.0	27.000	134.148
Y (ppm)	4.0	18.00	25.0	32.4	201.0	7.000	23.995
Zn (ppm)	16.0	85.0	104.0	126.0	638.0	21.000	102.567
Zr (ppm)	11.0	55.1	72.3	102.0	551.0	21.400	73.031
N (%)	0.03	0.24	0.34	0.47	2.21	0.110	0.337
TOC (%)	0.53	2.99	4.39	6.13	25.31	0.147	4.342

Q_1_, Med; and Q_3_ are the sample quartiles (25th, 50th and 75th percentile); MAD is median absolute deviation; g is geometric mean

1 $$ S^{D} = \left\{ { \left( {x_{1} , x_{2} , x_{3} , } \right. \ldots , \left. { x_{D} } \right) : x_{i } > 0 \left( i \right. = 1, 2, 3, \ldots , \left. D \right), \mathop \sum \limits_{i = 1}^{D} x_{i} = \left. \right\} } \right. $$

where κ is a constraint-sum constant; *x*_*1*_*, x*_*2*_*, x*_*3*_*,…, x*_*D*_ are components of the composition *x*; and 1, 2, 3,…, *D* are parts of the composition *x*.

The simplex can “unfold” in the Euclidean vector space only after the proper transformation of its components. Since the treatment of the closed data seriously interferes with the methods of traditional statistics, this transformation is mandatory in order to safely apply standard statistical techniques. From a number of transformations used in the literature, the centred log-ratio transformation (clr) of raw (compositional) data, originally proposed by Aitchison [3], is used in this work. The application of the centred log-ratio is held indispensable for CoDa processing in multivariate statistical methods such as DFA since the clr preserves the original distances between corresponding compositions and allows them to be handled in a straightforward way [22, 67]. Simultaneously, the singularity problem inherent to a clr-transformed covariance matrix can be circumvented allowing DFA to operate on its reduced form, that is, not relying on the full rank of covariance [17]. Since clr-transformed data represent unbounded real vectors in a real space, Mahalanobis distances (MD) remain invariant regardless of which component may be removed from analysis [4]. Conveniently, nonessential clr-transformed variables may be amalgamated (“other”) and removed from further analysis.

Clr-coefficients can be computed from the following expression:

2 $$ clr\left( x \right) = \left( {\ln \frac{{x_{1} }}{g\left( x \right)}} \right.,\; \ln \frac{{x_{2} }}{g\left( x \right)},\; \ln \frac{{x_{3} }}{g\left( x \right)}, \ldots , \ln \left. {\frac{{x_{D} }}{g\left( x \right)}} \right) $$

where *x*_*1*_*, x*_*2*_*, x*_*3*_*,…, x*_*D*_ are components of the composition *x* and *g(x)* represents their geometric mean.

#### Discriminant function analysis—the strategy

DFA is a powerful statistical tool for approaching a great number of numeric attributes such as, in this example, the geochemical compositions of soils developed on the karst bedrock.

This technique aims to reduce problems with organization, distinction, or comparison of the vast body of data to a scale providing clearer insight into the underlying geological and environmental controls. In addition, data processed in this way can develop a mapping quality that explains the relationship among the original variables more clearly.

The aims and principles of DFA are described in detail elsewhere (e.g., [18, 20, 61]) and have been explained repeatedly by the present authors in various geochemical and environmental studies [23, 26, 28, 34, 49, 50, 52, 53, 65]. It suffices to say in this paper that DFA is a multivariate method that is particularly effective in pursuing the major sources of between-group differences which, in this study, derive their origin from the accumulation of heavy metals and possibly harmful elements (PHE) in karst soils. To this purpose, a vast body of data (1247 soil samples) must be previously organized in a manner that provides the most effective relation between the soil geochemical signature and various facets of the soil immediate environment. The definition of grouping criteria is crucial in this respect since geochemical patterns in the sampling media, as a rule, always follow the bigger picture on a regional scale—geological, environmental and other systemic constraints prevailing in the investigated area (Croatian Dinaric region). Of necessity, these principles are autonomous with regards to the analysed variables (see, e.g., [61]). One of the most obvious standards suitable for the group characterization in the present case is the underlying geology (lithology). This characterization is based on earlier research work [28] that has proved profitable, emphasizing the strong geochemical contrast between the soil geochemistry of the two regions of Croatia broadly defined as the DIN and PAN areas. Consequently, although the bedrock is predominantly carbonate in both regions, bedrock underlying the soils of the DIN was expected to be lithologically sufficiently diversified to affect the geochemical signal. Further, earlier investigations [42] strongly suggested that geographical division (zoning) may show distinctive preferences in the areal distribution of certain elements irrespective of the underlying geology. Last but not least, the recent investigations concerning the GEMAS Project (Geochemical mapping of agricultural and grazing land soil) [60] indicated the usefulness of the land cover classes, borrowed from the Corine Land Cover (CLC) inventory, in the search for environmental impacts on the geochemical composition of soils.

**Fig. 3:**
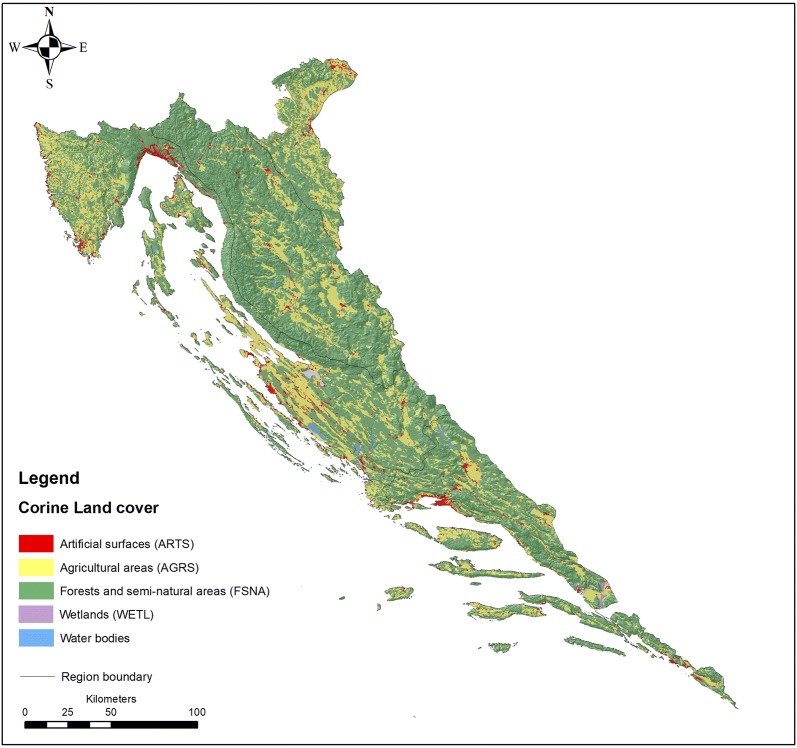
CLC map (the most general level of standard CLC classification, Label 1)

Following the suggestions given above, three main themes of this work are outlined with respect to the presented grouping strategy—GEOLOGY, REGION, and CLC. In each particular case, a different number of classes is derived depending on the nature of the grouping variables, which originate, at least partly, from the familiar ‘clorpt’ equation (climate—organisms—relief—parent material—time) that describes the role of variables (state factors) in the process of soil formation (e.g., [9, 54, 55]). This concept was later extended to include ecosystem, soil, vegetation and fauna (e.g., [10]) and finally reviewed in a recent work on soil complexity and pedogenesis [56]. The groups (Table 2) are formed according to the following sources: the GEOLOGY division is based on the general lithology of the investigated area accepted from the Geological Map of the Republic of Croatia (1:300,000; [14]) and contains five groups consisting of siliciclastic rocks (1), Quaternary sediments (2), carbonate rocks (3), carbonate clastic rocks (4) and flysch (5); the REGION division uses the map of agricultural regions and sub-regions of Croatia [8] modified at the DIN-PAN border to accommodate the distribution of predominant carbonate lithology and is composed of five groups consisting of North, Mid and South Mediterranean (NMED (1), MidMED (2), SMED (3)), mountainous (MOUNT (4)) and sub-mountainous (SubMOUNT (5)) regions; and finally, the CLC division exploited the most general level of standard CLC classification (Label 1) from the Corine Land Cover 2012 (CLC2012) raster data (European Environment Agency (EEA, http://www.eea.europa.eu)), combined into 4 groups consisting of artificial surfaces/urban or builtup areas (ARTS (1)), agricultural land (AGRS (2)), forests/forest land and semi-natural areas (FSNA (3)) and wetlands (WETL (4)) (Fig. 3). In all four cases containing 1247 valid objects in total (N), the same suite of variables (*p* = 28) is used.

**Table 2:** Grouping criteria

Group	GEOLOGY	*n*	REGION	*n*	CLC	*n*
G1	Siliciclastic rocks	47	North (NMED) Mediterranean	242	Artificial surfaces (ARTS)	61
G2	Quaternary sediments	98	Middle (midMED) Mediterranean	234	Agricultural surfaces (AGRS)	355
G3	Carbonate rocks	946	South (SMED) Mediterranean	313	Forests and (FSNA) seminatural areas	829
G4	Carbonate clastic rocks	103	Mountainous zone (MOUNT)	346	Wetlands (WTL)	2
G5	Flysch	54	Sub-mountainous zone (subMOUNT)	112		

*N* number of cases in each respective group

## Results and discussion

A concise summary of the main results of the analysis is displayed in the joint table (Table 4) comprising the three explanatory discriminant models. The overall significance of their discrimination is tested beforehand by the appropriate multivariate tests (Table 3), revealing the vanishingly low associated probabilities at the p < 0.05 level, which are essential in order to proceed safely with computing discriminant functions (DF). In virtue of the high separation potential of the computed DFs in all discriminant models, ample parsimony was achieved by attaching a plausible geological meaning to the selection of functions explaining the highest portion of the total variance. As shown in Table 4, most of the total between-group variance (80% or more) is sufficiently explained in all models by the first two DFs. Additionally, a grouping principle accountable for a high number of pre-defined groups has proved itself quite suitable for DFA analysis as the overall classification rate is rather high, amounting to a classification efficiency of 80% and greater in the cases of the REGION and GEOLOGY criteria, respectively (Table 5). It must be noted in this regard that raising the level of CLC degrades the classification rates remarkably, reducing the values from 70% for the first level (CLC-1) with four registered groups to approximately 50% for the second level (CLC-2) containing 11 registered groups, and finally to 33% for the third level (22 recorded groups). This situation is why the base level (CLC-1) is preferred from among the different choices for the purpose of this investigation.

**Table 3:** Multivariate test for overall significance of discrimination

	Models		
	GEOLOGY	REGION	CLC
No. of groups	5	5	4
Wilks’ lambda	0.404	0.075	0.760
Approximate F ratio	11.037	39.597	4.164
Degrees of freedom	[112; 4828]	[112; 4828]	[84; 3638]
p level	p < 0.000	p < 0.000	p < 0.000

**Table 4:** Tests of residual roots (discriminant functions) for all three models 3.3

DF	Eigen value	Eigen (%)	Eigen cum	Canon. R	Wilks’ λ	χ^2^	df	p-level
GEOLOGY								
1	0.558	51.94	51.94	0.598	0.404	1113.1	112	0.000
2	0.334	31.11	83.05	0.500	0.630	568.0	81	0.000
3	0.119	11.05	94.10	0.326	0.841	213.5	52	0.000
4	0.063	5.90	100.00	0.244	0.940	75.6	25	0.000
REGION								
1	2.462	58.52	58.52	0.843	0.075	3181.7	112	0.000
2	0.887	21.08	79.60	0.686	0.260	1654.7	81	0.000
3	0.509	12.10	91.70	0.581	0.491	874.0	52	0.000
4	0.349	8.30	100.00	0.509	0.741	368.1	25	0.000
CLC								
1	0.201	68.10	68.10	0.409	0.760	337.7	84	0.000
2	0.059	20.18	88.28	0.237	0.912	112.8	54	0.000
3	0.035	11.72	100.00	0.183	0.967	41.8	26	0.026

**Table 5:** Classification matrix

Observed groups	Predicted groups																			
	GEOLOGY							REGION							CLC					
	G1	G2	G3	G4	G5	Total	% correct	G1	G2	G3	G4	G5	Total	% correct	G1	G2	G3	G4	Total	% correct
G1	24	6	16	1	0	47	51.06	211	6	3	19	3	242	87.19	4	15	42	0	61	6.56
G2	7	24	52	5	9	97	24.74	10	183	18	22	1	234	78.21	7	120	226	2	355	33.80
G3	16	12	889	9	20	946	93.97	12	28	263	9	1	313	84.03	8	71	749	1	829	90.35
G4	0	2	79	20	2	103	19.42	11	12	7	288	28	346	83.24	0	0	0	2	2	100.00
G5	0	2	17	1	34	54	62.96	4	0	1	21	86	112	76.79						
Total	47	46	1053	36	65	1247	79.47	248	229	292	359	119	1247	82.68	19	206	1017	5	1247	70.17

Group labels (first column) in each division (criterion) in accordance with the Table 2

### Functional models—labelling the discriminant functions

The labelling of DFs is essentially a transfiguration of the structural (mathematical) into functional (process) models, which in this case are essentially geochemical. The technique of labelling discriminant axes is thoroughly described elsewhere, including an explanation of why scatterplots are used instead of biplots in the CoDa analysis (e.g., [23, 52, 65]). Suffice it to say that the group centroids (means) are exploited in this work as the alternative for the host of individual objects in the construction of the scatterplots. This alternative is used in order to improve the intelligibility of representation, which may be marred by a high number of sample points occupying the reduced discriminant space. The group means are also useful later in calculating the contribution of each DF to a particular group.

**Fig. 4:**
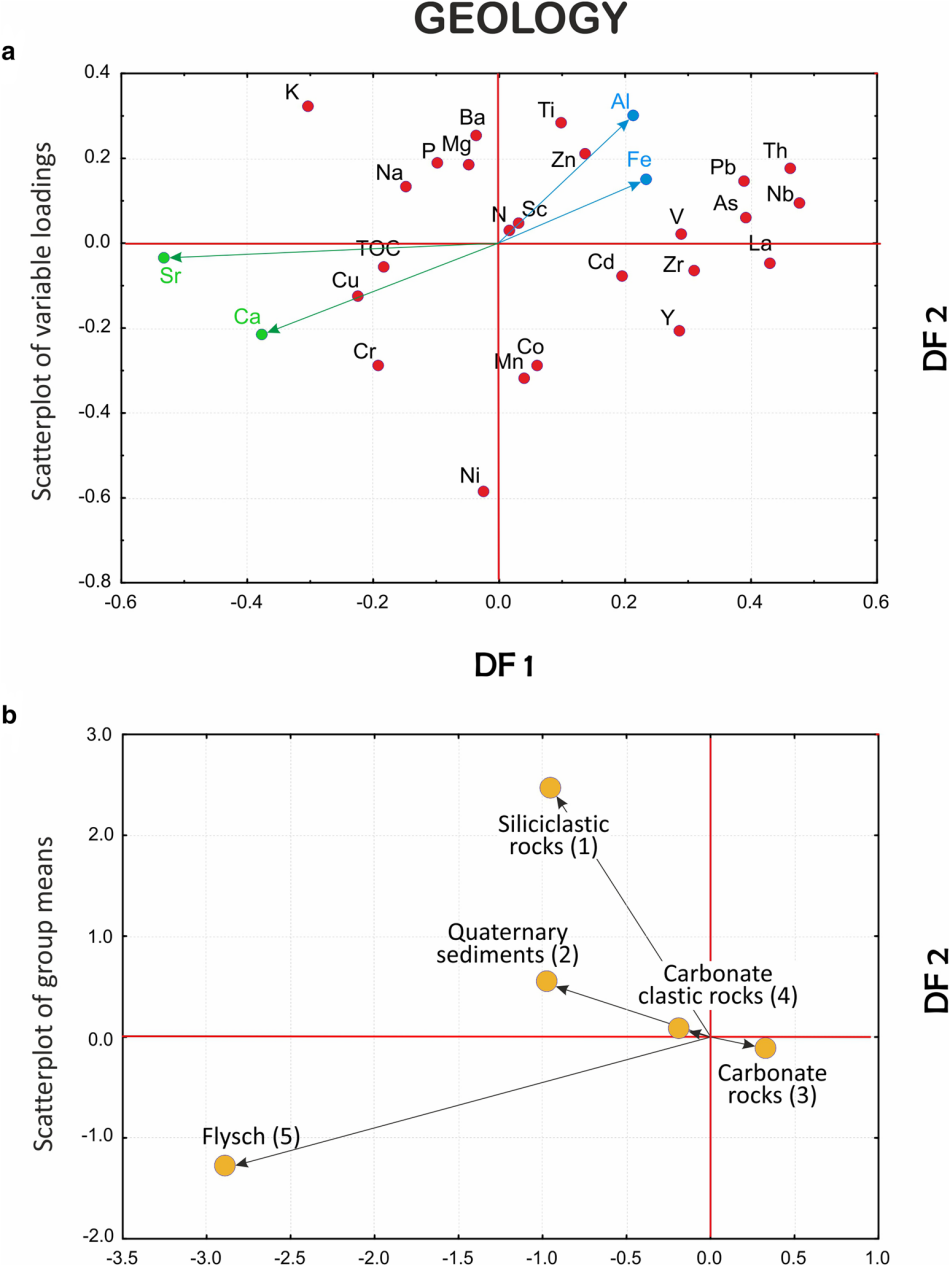
Comparison between variables and groups in the GEOLOGY, REGION and CLC discriminant function models (clr-transformed data): scatterplots of **a** variable loadings and **b** individual objects (samples) in the reduced discriminant space of the first two discriminant functions (DF1–DF2)

**Fig. 5:**
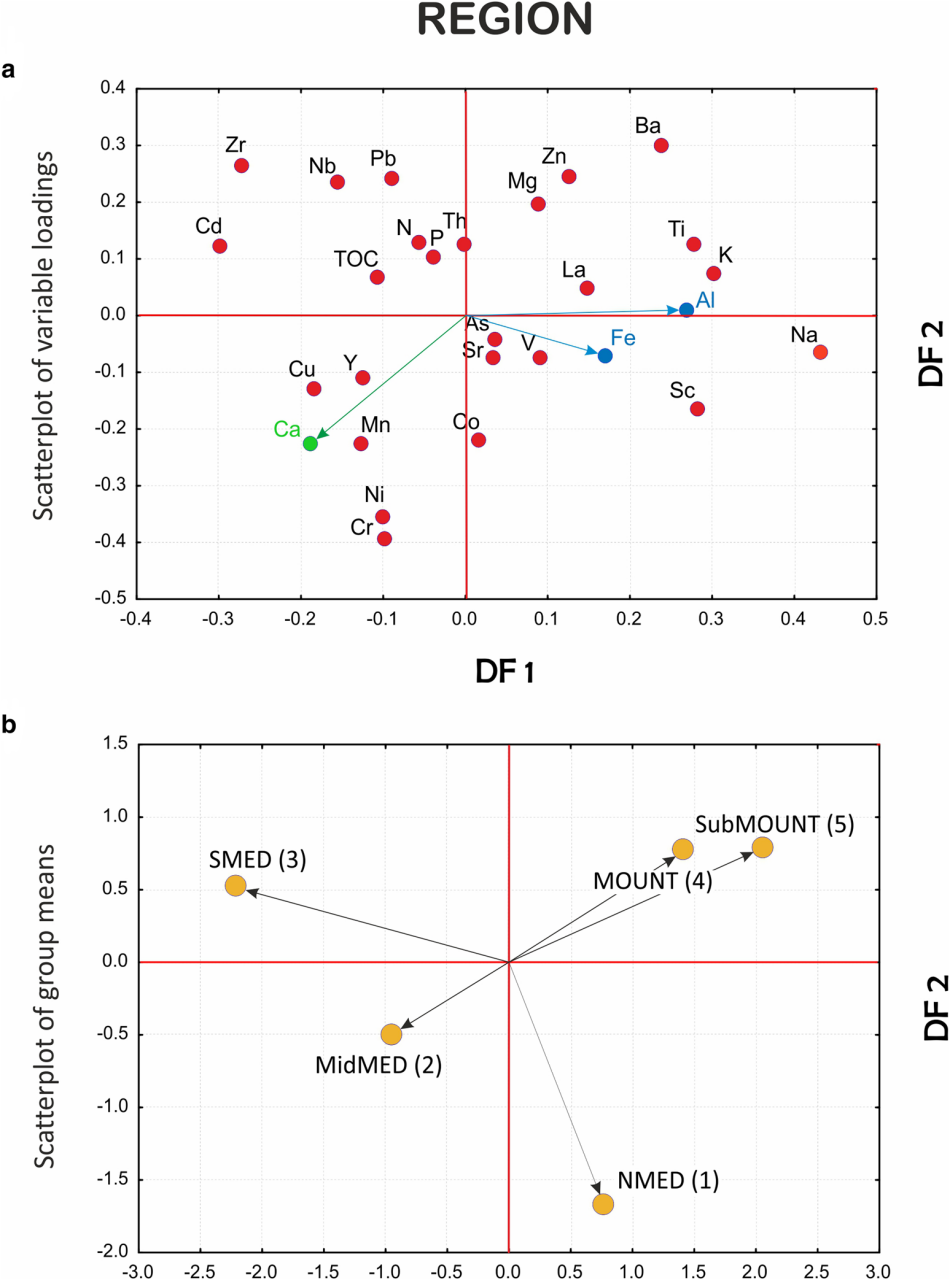
Comparison between variables and groups in the GEOLOGY, REGION and CLC discriminant function models (clr-transformed data): scatterplots of **a** variable loadings and **b** individual objects (samples) in the reduced discriminant space of the first two discriminant functions (DF1–DF2)

**Fig. 6:**
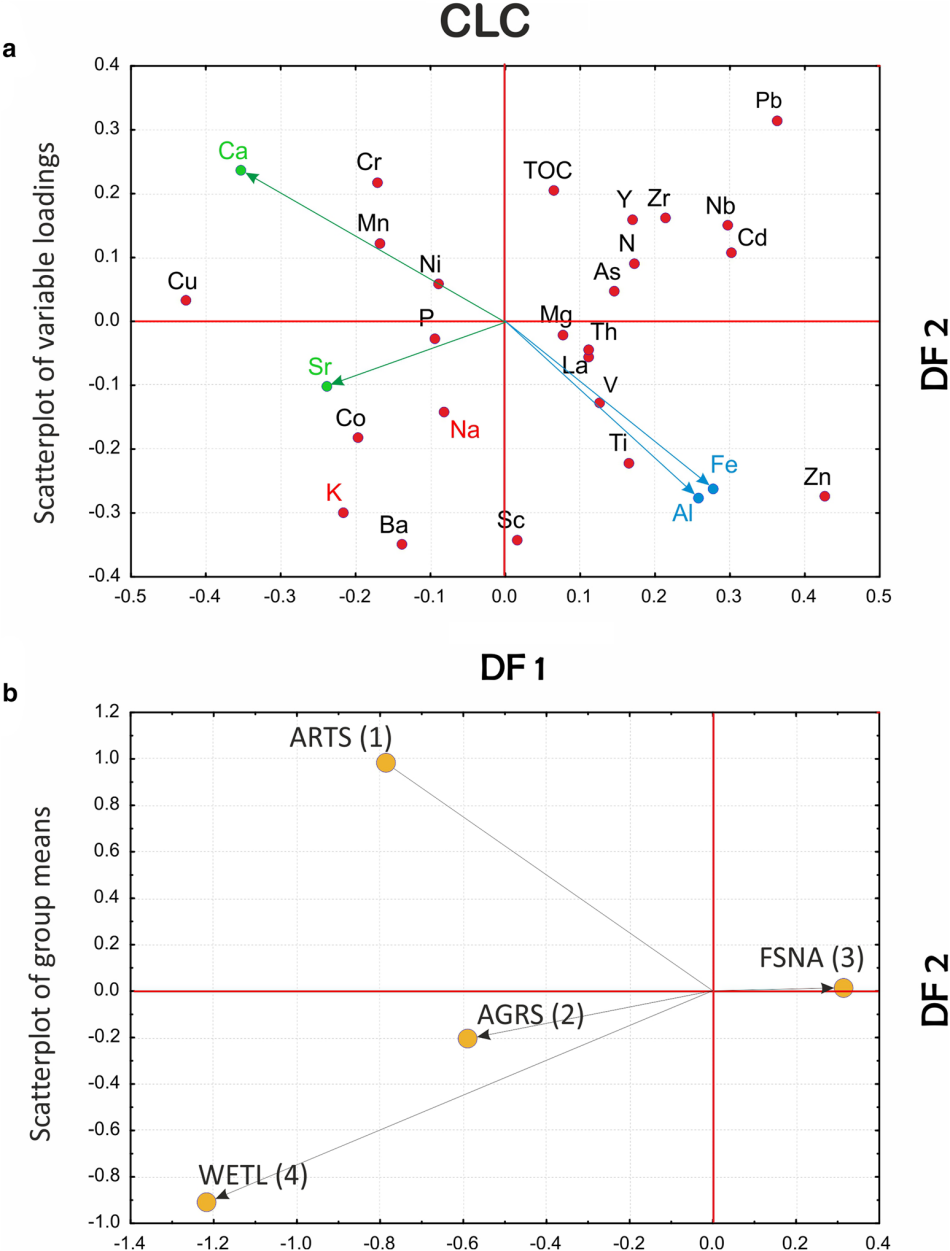
Comparison between variables and groups in the GEOLOGY, REGION and CLC discriminant function models (clr-transformed data): scatterplots of **a** variable loadings and **b** individual objects (samples) in the reduced discriminant space of the first two discriminant functions (DF1–DF2)

Scatterplots of variable loadings and group centroids are constructed for all discriminant models applying the first two DFs that explain the greatest portion of the between-group variance. The models are compared using multiple scatterplots of the DF1 and DF2 pairs of discriminant function (orthogonal axes) (Figs. 4, 5 and 6).

#### GEOLOGY model

In the GEOLOGY model (generally referring to the parent-material state variable, *p*) the first discriminant function DF1 separates on the basis of the carbonate/siliciclastic lithological contrast of the parent material and corresponding affinities of certain elements, principally Ca and Sr, identifying the flysch bedrock as the main source of geochemical variation in the soil samples (Fig. 4). DF1 is thus essentially monopolar, emphasizing the uniqueness of the flysch group, which plots far from the intersection of the DF1 and DF2 axes. This arrangement is essentially caused by the nature of the parent material as one of the crucial state factors (variables) of soil formation. Soils that evolved on flysch (mostly Leptosols (rendzinas)) and formed on soft marls and weakly consolidated calcareous sandstones are typically “immature”, that is, incipient and undeveloped as a result of the strong dynamism involving progressive and regressive pedogenesis in the process of rapid erosion and mixing of fresh parent material with the already formed regolith. This process, recognized on the Istrian Peninsula [40, 48, 51, 57, 58, 75] and elsewhere along the Adriatic coast [27], results in a “dilution effect” that places in clear relief the flysch- and carbonate-derived soils. As a rule, undeveloped (flysch-derived) soils do not exhibit typical enrichment in trace elements but stable or even depleted concentrations instead [38], while elevated contents of carbonate minerals (elevated Ca and Sr) are the result of poor drainage and leaching, which, conversely, is a norm of “mature” soils evolved over carbonate bedrock. Thus the latter, develop in a quite different pedo-environment, often end up as a repository for PHE and other trace elements whose accumulation may be additionally enhanced by human-influenced environmental processes [51].

Apart from DF1, whose primary discriminatory role is the flysch/carbonate bedrock contrast, DF2 adds another dimension to the model and explains the most of the remaining (residual) variance left after DF1 is removed. DF2 is also concerned with the flysch group, which is separated from the “siliciclastics” group (siliciclastic-derived soils) in a clearly displayed bipolar relationship. In this case, the flysch group differs from its clastic counterpart by reason of the Ni/K–Al–Ti–(…) inverse relationship revealing enrichment in Ni (followed by Co, Mn, and Cr, that is, potentially harmful elements, PHE) in the former and deficiency in the latter. On the other hand, the siliciclastics group is enriched in K and Al, most likely in the form of clay minerals and rock-forming feldspars, which are, conversely, relatively under-represented in the flysch-derived soils. The presence of the K–Al assemblage indicates in situ formation of soil clay minerals by alteration of aluminosilicate parent minerals which, simultaneously with illuviation, may be the dominant process of soil formation over the siliciclastic bedrock. Furthermore, the closeness of Fe and Al (Fig. 4) suggests the ubiquitous problem with soil acidity associated with soils developed on siliciclastic bedrock. Conversely, the PHE suite of elements in flysch-derived soils is probably of aeolian origin, accumulated relatively recently from the Raša port industrial zone and the Plomin thermal power plant in Istria [51]. The other three groups are clustered close to the DF2 axis, revealing their impartiality with regards to the geochemical signature of the overlying soils conveyed by this function. Indubitably, the central (near the axis intersection) position of the carbonate groups in both the DF1 and DF2 cases is induced by their excessive weight (84% of all observed or a priori classified data, Table 5) that, however, enables the uniqueness of the formerly described groups to be perceived in clearer relief. “Gravity” of the carbonate group is highlighted by the computed classification rates resulting in 94% correct assignments. A significant body of data (10.5%) has been relocated from other groups based on the mathematically predicted (a posteriori) classifications (Quaternary sediments and carbonate clastic rocks in particular) showing their greater affinity to the carbonate group, that is, the geochemical signature characteristics for carbonate-derived soils.

#### REGION model

The REGION model approximately adheres to the climate and relief as the state factors (*cl, r*) of soil formation. As in the former case, the first two discriminant functions are sufficiently informative in explaining the natural processes underlying the data structure (80% of the total variance). At first glance, the characteristic group pattern emerges showing SMED-MidMED-NMED group alignment with mountainous (MOUNT and SubMOUNT) groups apart in the hinterland, all mimicking the predominant northwest-southeast Dinaric direction of regional mountain ranges, albeit with the SMED and NMED groups in inverted geographical positions [cf. Fig. 1 (geographical position) and Fig. 5]. This peculiar diagonal arrangement needs clarification in both DF1 (SMED) and DF2 (NMED) domains. DF1 is bipolar and is primarily concerned with differences between the mountainous (MOUNT and SubMOUNT) and SMED soils, while DF2 shows differences between mountainous and NMED soils (Fig. 5). In the first case, elements forming the clay minerals such as Al, K and Na together with Ti, Fe, Sc and Ba are highlighted, a pattern suggesting the dominance of clay component and a possible role of Fe and Al oxy/hydroxides in sorption of PHE, especially Zn, in MOUNT/SubMOUNT soils (e.g., [45, 63, 71]). This interpretation is supported by the suggestive absence of characteristic trace elements such as Pb or Cd on the part of the latter in contrast to the soils from the southernmost coastal part of the investigated area (SMED). Additionally, SMED and MidMED soils are characterized by increased Zr and Ca, both indicating the presence of detrital heavy minerals such as zircon, external materials (of aeolian origin) [21, 73], and carbonate particles. These elements probably appear due to hindered leaching and eluviation on the characteristic carbonate lithology of undeveloped soils on flysch [51, 58]. The Ca/Al–Fe inverse relationship in DF1 reinforces the image of potential stress from Al and/or soil acidity in the MOUNT and SubMOUNT groups (Fig. 5).

DF2 is also bipolar, and it further clarifies the particular deployment of the two mountainous groups. Groups are separated in this case into the northern and central regional divisions (NMED and, less accentuated, MidMED) on account of increased contents of Cr, Ni, Co and Mn in the latter. These elements are typical PHE and pose great pressure on the natural ecosystem, unambiguously deriving their origin from anthropogenic sources represented by the numerous industrial and power plants and oil refineries in the upper Adriatic (Plomin, Rijeka) and metal processing factories in the middle Adriatic (Obrovac). The mountainous and sub-mountainous regions appear in this context as almost pristine areas except for Pb, which is also typical for the soils of the south Adriatic territory, probably for two reasons: long-range aeolian transport and high precipitation in case of the highest mountain areas and traffic in both regions. Last but not least, Ca is also among the elements associated with the SMED and MidMED groups (Fig. 5), emphasizing the NW–SE-trending increase in carbonate content in the topsoils.

#### CLC model

**Fig. 7:**
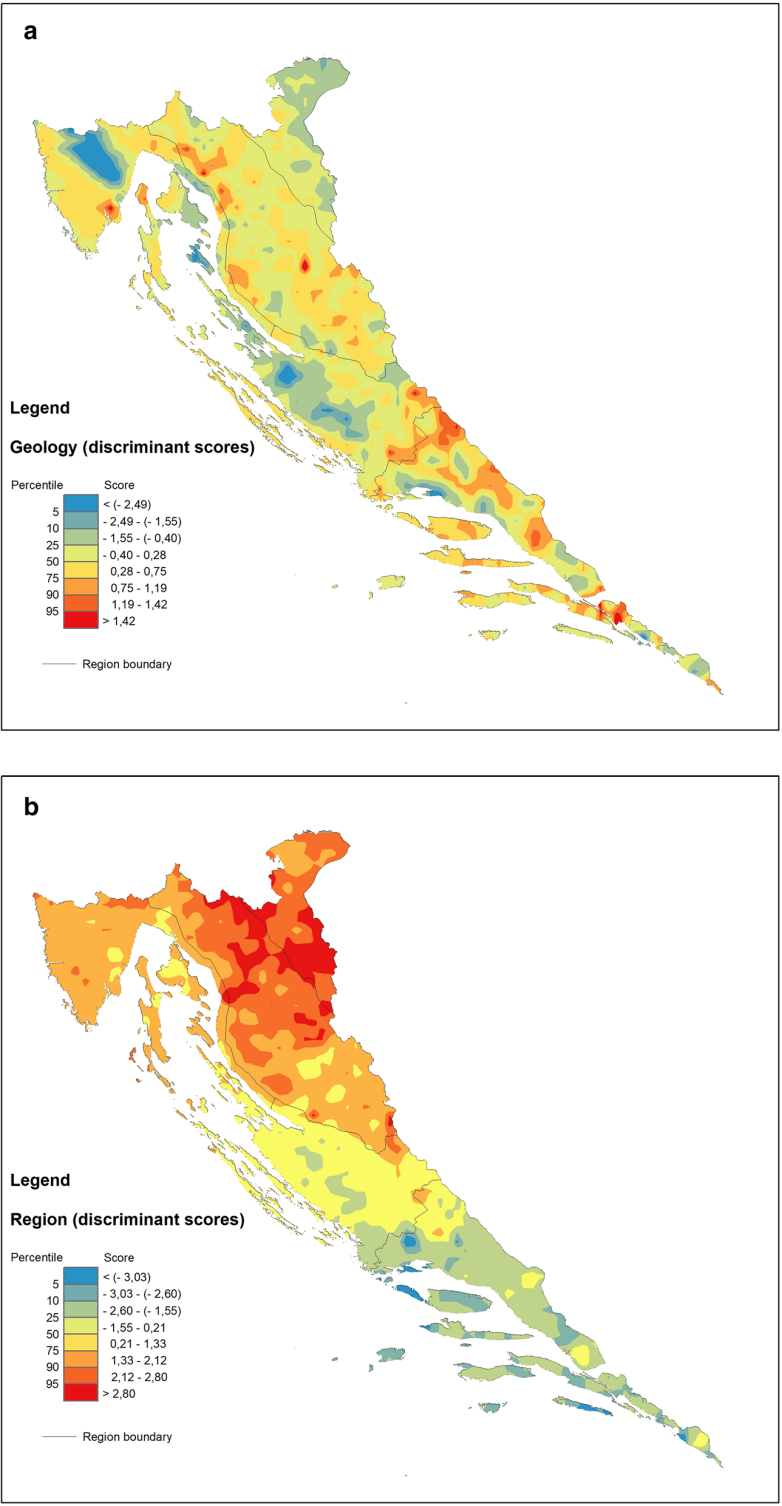

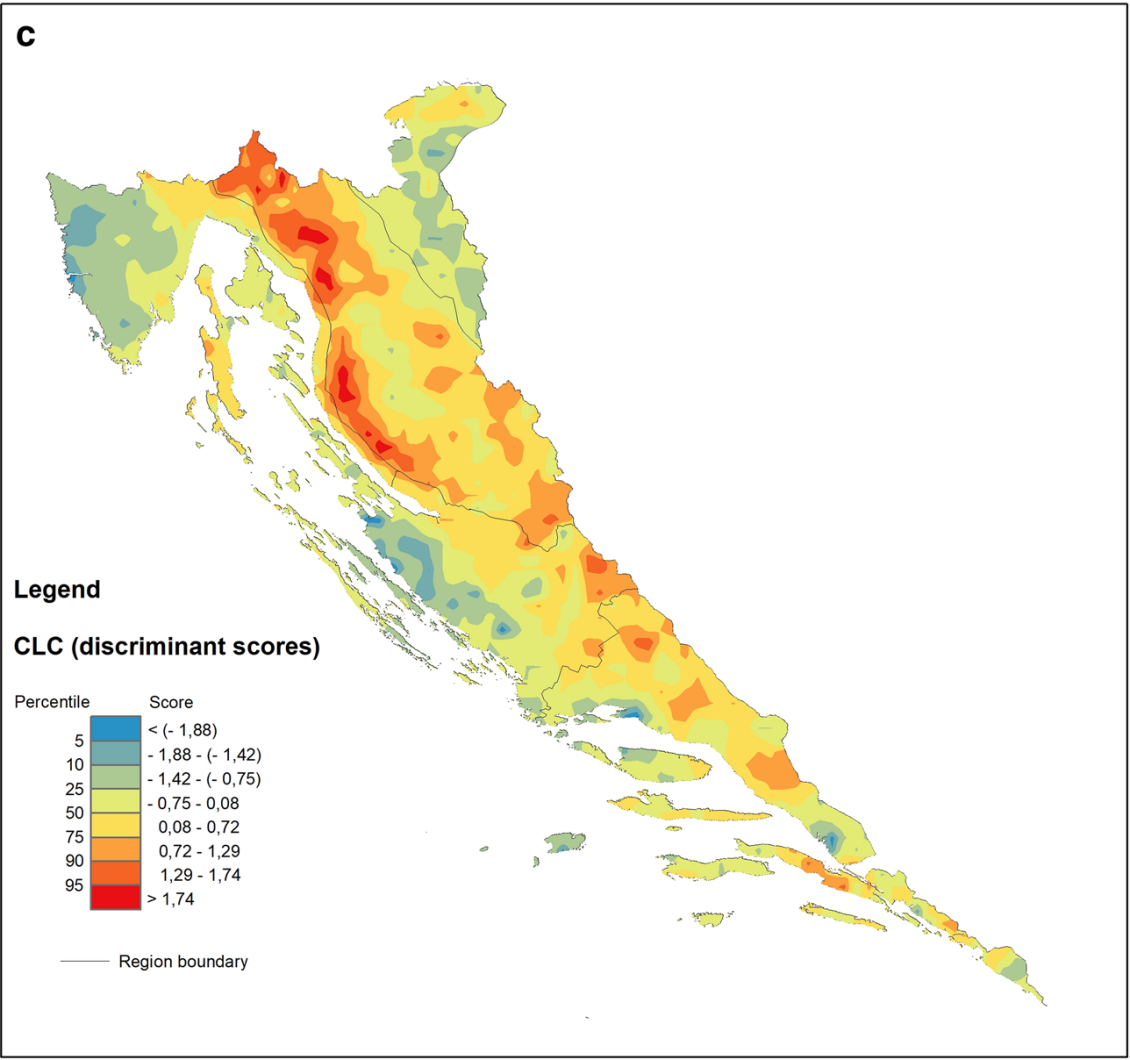
Discriminant score maps of **a** GEOLOGY, **b**, REGION and **c** CLC models representing areal distribution of the first (DF1) discriminant function. Increasing influence of the respective geochemical signatures displayed in warm colours (yellow–red)

The most general level of the CLC model, referring broadly to the ecosystem, vegetation and animal properties in Jenny’s extended soil functional-factorial model ([32], described in [10]), explains almost 89% of the total variability by the first two (DF1 and DF2) of three discriminant functions altogether (Table 3). The first of these is all-important (68%), and albeit bipolar, contrasting all first-level land cover classes against a single one—forest and semi-natural areas (FSNA)—it highlights the latter group which, similarly to the GEOLOGY model, gravitates to the centre of a scatterplot due to its extreme weight (over 66% of all data) with befitting 90% of correct a posteriori assignments (Fig. 6 and Table 5). Accordingly, all other groups are distinguished by their shared geochemical signal primarily lacking in those component parts that abound in the FSNA group. It comes as no surprise that FSNA in the explored model indicates that the forest ecosystem is under considerable environmental stress caused by acidic deposition and human interventions such as forest harvesting and agricultural activity. This problem is easily observed in the reciprocal position of Ca with respect to both Al and Fe resulting from increasing soil acidity (organic acids) (Fig. 6) [15, 25, 69]. Further, all other vital components also contribute to the gloomy picture of the impacted forest ecosystem showing deficiencies in clay component and soil fertilizers (K, Na and P) in the FSNA group with regards to the ARTS and AGRS groups and especially the WETL group (albeit the latter contributes merely two samples to the model), which are all relatively enriched in these elements. The close mutual positions of Al and Fe characterizing the FSNA group may well result from immobilization of organically bound Al and Fe due to precipitation, perhaps through the formation of solid Al–Si–OH, and Fe–OH phases in the coniferous forest soils [36] that predominate in the NW part of the mountainous Dinaric hinterland. Simultaneously, the presence of Pb, Cd and Zn, most likely deriving from acid rains in the elevated areas (DF2 in Fig. 5), only enhances the process of nutrient depletion and the accompanying contamination of the forest soils. On the whole, the buffering capacity of the forest soils against acidification is lower with respect to agricultural or otherwise used soils (ARTS) due to liming or other acid-neutralizing amendments [5]. To this feature must be added the problem of long-recognized chronic nitrogen deposition via atmospheric pollution resulting in N-saturation in the forest topsoil [1] (see DF1 in Fig. 6). Naturally, the relatively increased carbonate component together with the K–Na–P suite in other groups not only may result in a negative image of FSNA but also may emerge through anthropogenic impact (fertilization) that is intense in some areas (including Cu for vineyards, as on the Istrian Peninsula) (see Fig. 7c).

As for DF2, it provides additional insight (20%) into the group deployment separating ARTS from WETL based primarily on the high contrast associating Pb with artificial surfaces with regards to the latter. However, due to its characteristic geochemical signal, the WETL group with its mere two samples is not confounded with any other group, let alone ARTS, which on the contrary loses almost all of its objects (94%) to other groups, seriously questioning its a priori defined integrity in the investigated area. As seen from Table 5, exactly the FSNA soils accepted the majority of ARTS samples. The AGRS group with only 34% of correct assignments is also almost imperceptible as a standalone group losing the majority of its samples to FSNA. Thus, precisely the latter group profoundly characterizes the geochemical signature of the dominant land cover type in the study area, greatly altering the original CLC map (cf. Figs. 6 and 7c).

### Functional models—soil geochemical maps

The key feature of DFA modelling is that it produces numeric values (discriminant scores) suitable for spatial display of parameters accounting for discrimination of investigated groups. Hence, such modelling indirectly expounds both dissemination of the group samples and internal cohesive strengths of groups on the terrain. Concerning the latter, the models also provide estimates announcing how closely the group samples hold together by virtue of the probability that any case (sample) holds on to a particular group (via posterior classification probabilities) and thus ultimately highlighting the processes (explained by the predictor variables) that account for a particular spatial pattern in the investigated area. Geochemical maps generated in this way may be very helpful, for example, in physical planning because they promptly indicate the quarters of adverse impacts on the environment produced by human activity. Karst terrains are especially vulnerable in this case, and forest ecosystems with the increasing problems of acidification, soil erosion, disruption of the water cycle and possible loss of biodiversity are particularly so. Statistically speaking, their profits heavily rely both on success rates calculated in the overall classification design and on the power of discrimination functions to distinguish among groups with the highest accuracy possible. Accordingly, two types of geochemical maps are constructed in this work based on two different families of statistical indices generated by DFA, namely, the maps of posterior (post hoc) probabilities (regarding the specific group selected on the basis of its specific relevance) and the maps of discriminant scores (with respect to a particular DF). Both categories have already proved useful in various geochemical and environmental investigations [28, 65].

#### The map generation

The maps are generated using the ArcGIS™ 10.2.1 extension Spatial Analyst with the Universal Krigging method. For the purpose of map generation, the discriminant scores are divided into eight percentile classes: 5th, 10th, 25th (lower quartile), 50th (median), 75th (upper quartile), 90th and 95th percentiles because the application of the same percentiles for all data allows comparison of the respective spatial distribution maps. Maps of posterior probabilities are divided into seven probability classes: < 0.10, 0.10–0.25, 0.25–0.50, 0.50–0.75, 0.75–0.90, 0.90–0.99 and > 0.99. In the case of posterior probability maps, percentiles are provided by the spreadsheet containing posterior probabilities generated during the computational process. The classes displayed on the geochemical maps range in colour from blue hues for the lowest via green, yellow, and orange to red for the highest values.

In the former case, classification efficacy serves as a powerful indicator by which the stability of the previously defined groups can be screened, weighing mathematically predicted against original (observed) classifications (Table 5).

#### Discriminant function vs. posterior probability maps—mapping the soil processes and validation of grouping criteria (classification rates)

Inspection of the plots showing the most informative discriminant functions (DF1 and DF2) allows cross-comparison between the models—an approach elucidating the dominant processes in control of the geochemical signature in the soils of the investigated area (Figs. 4, 5 and 6). Thus, it is readily apparent that the combination of certain elements such as Al, Fe, K, Na, Zn, Pb, and Ti signalling the presence of organically bound metals, clays and some potentially harmful elements (PHE) is regularly affiliated with particular groups in all models. This arrangement bonds siliciclastic rocks (GEOLOGY), mountainous zones (REGION), and forest and semi-natural areas (mostly woodlands, CLC) into the sphere of influence controlled by the processes that endanger the karst region at large, especially the forested hinterlands behind the coastal mountain areas. The range of the discriminant scores is displayed on the respective discriminant score maps, which can be unambiguously interpreted on the single process basis (after a selected DF). On the other hand, posterior probability maps are understood differently since their orientation is towards validation of the group integrity. Accordingly, they are designed on a single group basis, highlighting the particular group as reflecting the sum of all relevant processes (represented by respective DFs) that affect its cohesion in the investigated area, albeit each with a different contribution. The involvement of individual DFs in each individual group can be easily calculated from the relative position of the group centroid represented by its discriminant scores (group mean) on all computed discriminant functions. Note that in the case of only two discriminant functions (reduced discriminant space represented by DF1-DF2 axes on 2D scatterplots, Figs. 4, 5 and 6), this influence is specified by the distance (vector length) of the group (G) from the origin (DF1/DF2 intersection) in a simple Pythagorean relation. In a multi-function case, the length of the respective group vector is extended accordingly in n-dimensional discriminant space and can be displayed by the following equation:

3 $$ {\mathbf{G}} = \left| { \left( {DF1, DF2, \ldots , DFn} \right)^{T} } \right| = \sqrt {DF1^{2} + DF2^{2} + \cdots + DFn^{2} } $$

where DF1, DF2, …, DFn denote the coordinates (discriminant scores, i.e., point projections onto the axes) of a particular group centroid (G). The allowance of each DF for a group is then computed as DF(n) = DFn^2^/G^2^.

**Fig. 8:**
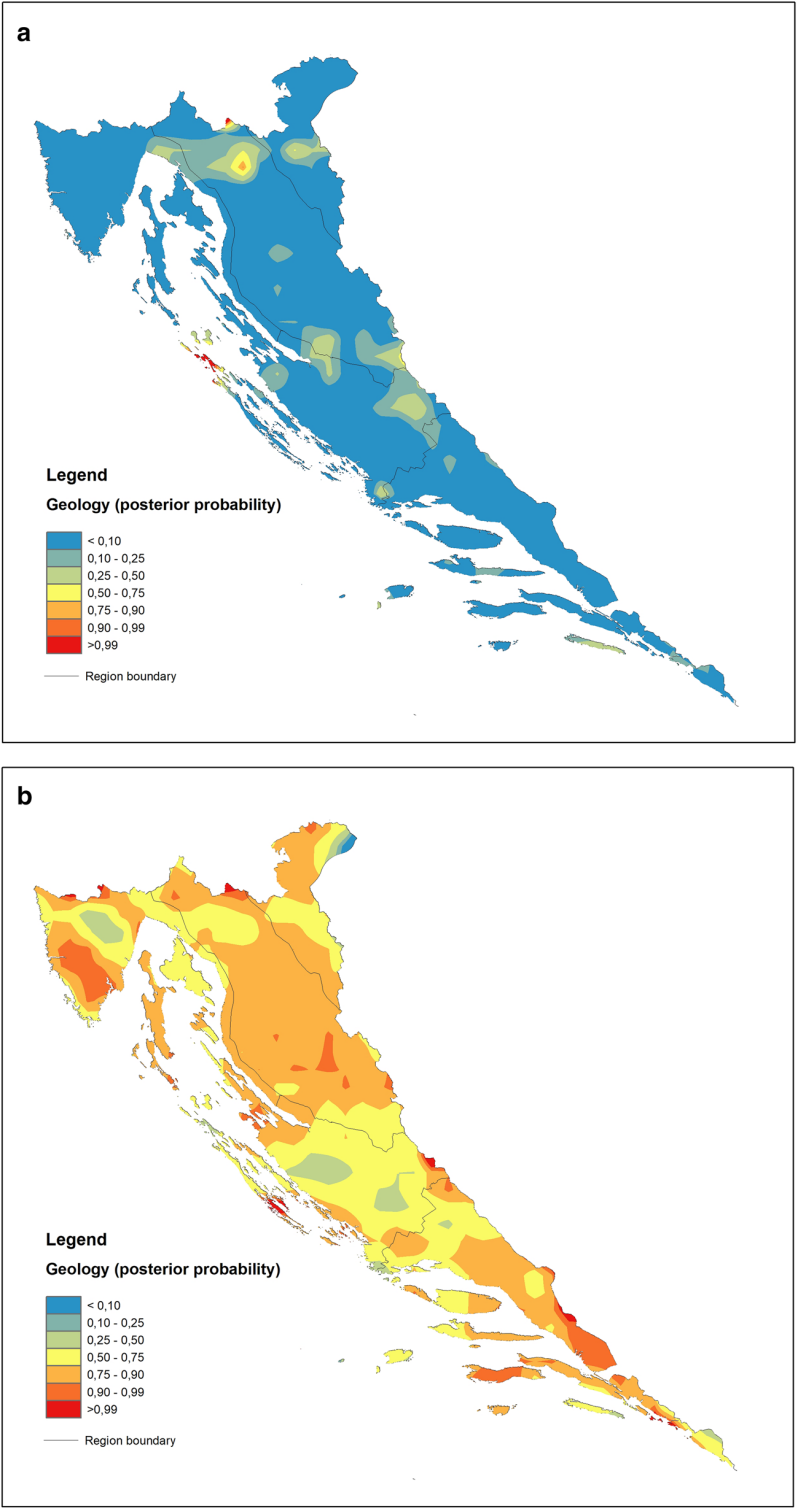
Maps of posterior probabilities of GEOLOGY model representing areal distribution of posterior (post hoc) probabilities computed for: **a** siliciclastic rocks (group 1) and **b** carbonate rocks (group 3). Increasing influence of soil geochemical signatures developed on siliciclastic (**a**) and carbonate rocks (**b**) displayed by the 75–100 percentile range (orange-red); increasing influence of other rock types (combined posterior probabilities of groups 2, 4 and 5) displayed by the 0–25 percentile range (green–blue). Zone of the mixing influences (yellow) displayed by the 25–75 percentile range

In the case of the GEOLOGY model, DF1 is focused on soil maturity, the property that strongly delineates the zones of flysch development within the karst environment (Central Istria and the North Dalmatian hinterland in particular, blue hues on Fig. 7a), which are characterized by soils largely containing properties inherited from the parent rocks and thus being rich in carbonate material (Ca and Sr). Warm hues display the transition towards carbonate rocks (groups 3 and 4) (Fig. 7a) via siliciclastic rocks (1) and Quaternary sediments (2) (Fig. 4), as indicated by the decrease in carbonates and increase in elements of poor mobility under all environmental conditions such as Th, Nb and La due to the high stability of the hosting minerals (oxides and silicates). This pattern, characteristic for “mature” soils evolved on carbonate bedrock (especially in coastal mountainous ranges), has been recognized in earlier investigations and is highly perceptible on mono-element geochemical maps produced as a result of the geochemical mapping of Croatia [27]. However, from the group perspective, the GEOLOGY model may yield additional information exposed in a post hoc map constructed for a single group. This procedure is advantageous in a sense that it may bring to the fore the most prominent process underlying all models irrespective of the proposed grouping criteria, not necessarily represented by the first function (DF1). If the latter be the case, the differences between the maps might prove insignificant from the standpoint of the post hoc classification of carbonate rocks (3) (cf. Figs. 7a and 8b). On the other hand, all models exhibit characteristic cross-pollination with regards to the Al–Fe–Zn–Ti cluster that is characteristic of certain groups—siliciclastic rocks (1) in the GEOLOGY model, mountain (4) and sub-mountain (5) regions in the REGION model and forest and semi-natural areas (3) in the CLC model. In GEOLOGY, the “correct” classification rates (p > 0.5) are almost exactly limited to outcrops of siliciclastic rocks (1) appearing in the interior parts of Croatian karst (warm hues on Fig. 8a). The DF2 signalling acute soil acidity is prominent in this group, with an over 82% contribution among the model DFs (see the scatterplot of group means, Fig. 4). The influence of DF1 (maturity) in the flysch group (5) is exactly the same (DF2 is only 16%), the case already recognized from the map of discriminant factor scores (Fig. 7a).

**Fig. 9:**
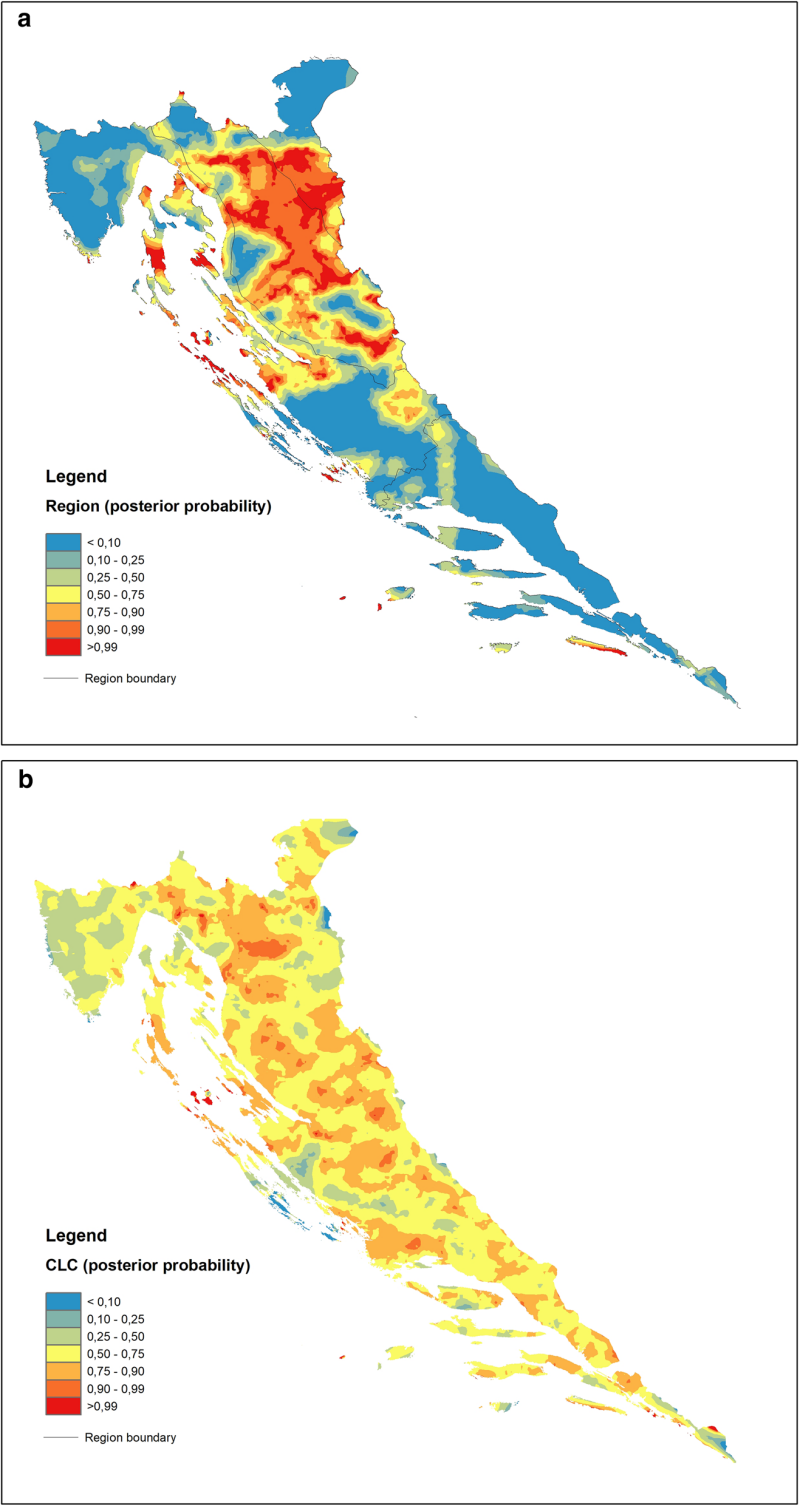
Maps of posterior probabilities of REGION (**a**) and CLC (**b**) models representing areal distribution of posterior (post hoc) probabilities computed for combined MOUNT + subMOUNT groups (groups 4 and 5) in the former, and FSNA (group 3) in the latter. Influences of respective soil geochemical signatures expressed through the posterior probabilities same as in the Fig. 8

The REGION model exhibits sharp delineation between the groups with regard to the changing influence of elements loading on DF1 in the NW–SE direction. There is a characteristic “neutral” geochemical signal characterizing the NMED and MidMED groups (Istria and North Dalmatia with their interiors) set within the interquartile range (25th–75th percentiles) (Fig. 7b). In “regional” terms, this is the area where the Na–K–Al–Ti–Sc–Ba–(Fe) vs. Zr–Cd element clusters are well-balanced, leaving the MOUNT and SubMOUNT areas on one side and SMED on the other as “outliers”. Accordingly, the former groups seem most endangered by the effects of soil acidity (Al toxicity) while the latter, for its own part, suffers increased anthropogenic inputs of Cd and Cu as well as Zr as a mark of residual soil evolved on karst bedrock. While copper is most likely directly related to viticulture developed in the southernmost part of the Croatian coastal area (Adriatic), cadmium in the SMED topsoil may partly originate from the former metal industry and former agricultural use of poor-quality fertilizers (Neretva valley). From the group standpoint (Fig. 9a), the correct assignment of samples to the MOUNT (4) and SubMOUNT (5) groups taken together (80%, Table 5) corroborates the close relationship between the distribution of mountain soils and the extent of acidification previously described. This process, represented by DF1 in the REGION model, participates with 67% in the MOUNT group and 56% in the SubMOUNT group. However, it also reveals that parts of the north Dalmatian area (MidMED) and north Adriatic islands (NMED) may experience the same problem as interior mountain areas (warm hues on Fig. 9a) being post hoc reclassified as SubMOUNT/MOUNT soils according to their geochemical signature. The sharp WSW-ENE line dividing these groups from “non-mountain” groups (MidMED and SMED) to the southeast in all probability represents the line dividing depositional environments on the east Adriatic coastal area during Pleistocene and Holocene, namely, the northern (Po River) and southern (South Adriatic) provenances ([70] and references therein). The northernmost part of the map is also excluded from the scheme, challenging the pre-established idea of some interior mountains such as Medvednica and Žumberak Mts. as parts of the integral Croatian karst territory (Dinaric) and relocating them (from the REGION-model perspective) into the Pannonian instead [28].

The CLC model most of all directly combines the overt Al–Fe soil toxicity with contamination by PHE (Zn, Pb and Cd in particular), seriously affecting the FSNA (3) on carbonate bedrock (Fig. 6). Thus, damage occurs to FSNA as an ecosystem, which is most evident on the coastal mountains of the north Adriatic zone (Fig. 7c, warm hues). Coastal ranges represent the first front of wet (acid rains) and dry (aerosols and gases) deposition caused by the atmospheric emissions from burning fossil fuels in the onshore power plants. The second front is less affected, while some flat areas on the coast (Istria and North Dalmatia) and in the transition zone towards the Pannonian area seem almost unaffected. From the grouping perspective, the FSNA (3) is the most seriously stricken by this process. As a group occupying the major part of the investigated area (60%), it is also the group with the greatest number of correct assignments (90%, Table 5) and with practically full participation of DF1 in its development and areal distribution. The predominance of other groups carrying different geochemical signals is focused on bordering areas such as Istria and northern and southern Dalmatia, as well as the farther north (Fig. 9b), a trait that is well-matched with the distribution of DF1 displayed on the CLC map of discriminant scores (Fig. 7c).

## Concluding remarks

In this work, a comprehensive investigation of the geochemical composition of topsoils developed in the Dinaric part of Croatia (DIN) was performed, with the purpose of elucidating the underlying mechanisms controlling the mobility and variations in PHE distribution perceived from various environmental perspectives, notably, the geologic setting, regional placement and diverse land use. The latter were employed in discriminant function analysis in place of grouping criteria for the analysed objects (sampled soils), independent as regards the soil geochemistry and approximately corresponding to the state factors in the familiar ‘clorpt’ equation for soil evolution. Three distinctive discriminant models emerged from the analysis disclosing the complex relationships among observed geochemical data, each with its own set of discriminant functions, namely, GEOLOGY, REGION and CLC. Albeit a number of multi-element soil geochemical signatures typical for investigated environmental domains were isolated, a particular geochemical signal was highlighted in all models, namely, the Fe–Al association related to siliciclastic bedrock, mountain areas (both MOUNT and SubMOUNT groups), and forest and semi-natural areas (FSNA). This result underlined the environmental challenges posed by soil acidification in the entire Dinaric karst area, though not necessarily by mobilizing the largest part of the variance in all models: acidification was the primary issue (DF1) in the models of REGION and CLC but only the secondary issue (DF2) in the case of GEOLOGY, where DF1 recognized Al–Fe clustering (along with the clay component) in soils derived from all lithologies rather as a mirror image (deficiency) of the carbonate (Ca and Sr) component that is, on the contrary, accumulated in flysch-derived soils. The main theme of the REGION model was discrimination between the soils from the NW part (MOUNT, SubMOUNT and NMED) and those from the SE part of the Croatian karst area (MidMED and SMED) based on the Ca/Al–Fe opposition as an indicator of Al and acidity stress in the former. The same image emerged in the CLC model, separating the “unexploited” areas (FSNA) affected by the same concerns from the other land-use types (AGRS, ARTS and WETL).

Two types of soil geochemical maps were constructed in the work, explicit discriminant function and posterior probability maps, in order to map the dominant (single) geochemical process (represented by DF1 in each model) and to check the integrity of a particular á priori defined group in the investigated area (map as a multi-function model). With remarkable accuracy, the first type follows the original grouping criteria, highlighting the areas where the performance of the mapped function (process) is the highest or the lowest. This distinction is particularly manifested in the case of the REGION DF1 model map, where the geographical division (as a grouping criterion) and the spatial distribution of DF1 match almost perfectly. Additionally, the GEOLOGY DF1 model map distinctly delineates the flysch outcrops, especially in Istria, while the CLC map draws attention to the heavily forested areas (FSNA) occupying the high mountains towards the coast hinterland (Velebit Mt. and Gorski Kotar area). The second type of map strongly depends on the correct post hoc group assignments, which is why in accordance with the group sizes, some “scavenging” may appear towards the smaller groups such as in the cases of the MOUNT + SubMOUNT posterior probability map where soils developed in the north Adriatic islands assume the characteristics of mountainous soils. On the other hand, the siliciclastic rock posterior probability map is an outstanding example of a highly cohesive group strictly under geological constraints (geologic bedrock) and controlled by the process defined by DF2 in the GEOLOGY model.

## Data Availability

The data used in this paper are available in the Geochemical Atlas of the Republic of Croatia [27] which is on the web site of the Croatian Geological Survey at http://www.hgi-cgs.hr; organic carbon data are available on the Croatian Environmental and Nature Protection Agency http://www.haop.hr/hr/pristup-informacijama.
